# New Insight into the Colonization Processes of Common Voles: Inferences from Molecular and Fossil Evidence

**DOI:** 10.1371/journal.pone.0003532

**Published:** 2008-10-29

**Authors:** Christelle Tougard, Elodie Renvoisé, Amélie Petitjean, Jean-Pierre Quéré

**Affiliations:** 1 UMR CNRS/uB 5561 Biogéosciences-Dijon, Université de Bourgogne, Dijon, France; 2 UMR 1062, INRA, Centre Biologie et Gestion des Populations, Campus International de Baillarguet, Montferrier/Lez, France; Max Planck Institute for Evolutionary Anthropology, Germany

## Abstract

Elucidating the colonization processes associated with Quaternary climatic cycles is important in order to understand the distribution of biodiversity and the evolutionary potential of temperate plant and animal species. In Europe, general evolutionary scenarios have been defined from genetic evidence. Recently, these scenarios have been challenged with genetic as well as fossil data. The origins of the modern distributions of most temperate plant and animal species could predate the Last Glacial Maximum. The glacial survival of such populations may have occurred in either southern (Mediterranean regions) and/or northern (Carpathians) refugia. Here, a phylogeographic analysis of a widespread European small mammal (*Microtus arvalis*) is conducted with a multidisciplinary approach. Genetic, fossil and ecological traits are used to assess the evolutionary history of this vole. Regardless of whether the European distribution of the five previously identified evolutionary lineages is corroborated, this combined analysis brings to light several colonization processes of *M. arvalis*. The species' dispersal was relatively gradual with glacial survival in small favourable habitats in Western Europe (from Germany to Spain) while in the rest of Europe, because of periglacial conditions, dispersal was less regular with bottleneck events followed by postglacial expansions. Our study demonstrates that the evolutionary history of European temperate small mammals is indeed much more complex than previously suggested. Species can experience heterogeneous evolutionary histories over their geographic range. Multidisciplinary approaches should therefore be preferentially chosen in prospective studies, the better to understand the impact of climatic change on past and present biodiversity.

## Introduction

Since the birth of phylogeography 20 years ago [1], a large number of molecular studies have investigated the influence of Quaternary climatic changes on the distribution and evolution of modern fauna and flora [2]–[7]. In Europe, a number of general evolutionary scenarios have been identified for certain species of temperate plants and animals: (i) the postglacial colonization of northern regions from Mediterranean refugia (Iberia and Balkans/Greece); (ii) the isolation of Italian populations due to the Alpine barrier; and (iii) the occurrence of four main suture-zones where populations from different refugia meet [3]–[5]. Several studies based on molecular and/or fossil data have challenged the universality of postglacial colonization from southern glacial refugia for a wide range of European temperate species [8]–[17]. Distinctive mitochondrial DNA (mtDNA) haplotypes indicate that Mediterranean regions were hospitable to some small mammals, insects and plants [8], [13], [18]. These regions may therefore have been a “hot spot” of endemism rather than a major source area for postglacial colonization [8]. Colonization may have occurred from cryptic northern glacial refugia, in for example Central Europe and Western Asia [7], [8], [11], [15]–[17], [19]–[22]. The northerly survival of species could thus be related to biogeographic traits (past and present-day geographic distribution, habitat preference, body size, mobility, generation time) [17]. Although the genetic divergence between the lineages of most European temperate species often predates the Last Glacial Maximum (LGM; 0.023–0.015 Myr), intraspecific phylogeographic reconstructions are generally explained as a relic of the LGM [3], [23]. The lack of phylogeographic patterns in European temperate mammals before the LGM could result from the erosion of the phylogeographic structure by population migration and mixing between the penultimate glacial period (0.300–0.130 Myr) and the LGM [23]. But what happened between the first appearance of European temperate species and the LGM?

**Figure 1:**
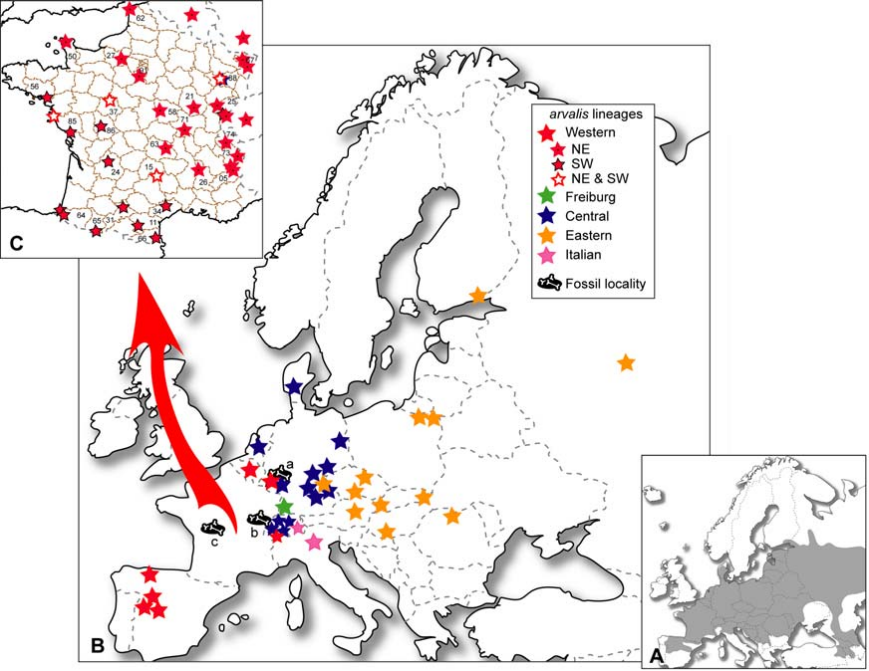
Geographic distribution of *Microtus arvalis* populations and fossil localities. The shaded zone (A) corresponds to the distribution range of the common vole [25], [31]. European (B) and French (C) populations are listed in Table S1. Fossil localities studied are: (a) Miesenheim I, Germany [29]; (b) la Baume de Gigny, France (Gigny, Jura) [49]; (c) le Taillis des Coteaux, France (Antigny, Vienne) [52]. Numbers are French zip codes.

The common vole (*Microtus arvalis*) is a small European rodent often considered a pest, although as with most Arvicolinae, it is a good species model for the study of evolutionary questions. This is because of its short generation time, good fossil record, well-known ecology and fast mtDNA substitution rates [24]–28. This vole has been present in the fossil record since the Late Cromerian (0.500–0.450 Myr) [29], [30]. It is currently widespread in Europe with a continuous distribution from the Atlantic coast of France to central Siberia, ranging in altitude from sea level to 3000 m in the Alps (Figure 1A) [25], [31]. Its range is limited by a double climatic barrier to the north by mean July temperatures <+16°C, and to the south by arid environments [32]. This vole builds tunnel systems connected by surface runways, and lives in open field areas (*i.e.* cultivated fields, temporary alfalfa and clover meadows and mountain grassland) [25]. The phylogeography of the common vole has previously been investigated from mtDNA control region (CR) and cytochrome *b* gene (cytb) sequences and microsatellites [6], [33], [34]. This rodent displays a clear phylogeographic structure with five evolutionary lineages identified over its range: Western (W), Eastern (E), Central (C), Freiburg (F) and Italian (I) lineages. Haynes *et al.* [6] agree with the general evolutionary scenarios of colonization (*e.g.* spread from different southern LGM refugia), whereas Fink *et al.* [33] and Heckel *et al.* [34] see things somewhat differently. For them, genetic diversity through Europe suggests glacial survival of the common vole outside the classical refugia and a potentially more ancient colonization (pre-LGM) from the northeast to the southwest of Europe. Nevertheless, neither the exact location of the *M. arvalis* lineage origin, nor the position of glacial refugia, or even the date of the colonization onset have yet been established. Fossil data have perhaps not yet been accorded the place they deserve.

The importance of combining molecular and fossil data to obtain more reliable interpretations of the spatio-temporal colonization processes has recently been underlined for other species [13], [17], [35]–[38]. Here, we consider the crucial parameters necessary for a complete description and understanding of such processes for the common vole. First and foremost, the molecular sample size was increased in order to try and improve the tree topology and robustness. This would allow clarification of the hypothetical implications of European southern and/or cryptic northern refugia in postglacial colonization. Mandible and tooth remains are commonly found in European palaeontological and archaeological sites [30] and this considerable fossil record was put to good account. Peculiar care was taken over the choice of fossil sites to avoid possible controversy about species determination (*M. arvalis* vs *M. agrestis*). Biogeographic traits (past and present-day geographic distribution, habitat preference and life-history traits) were also integrated in this multidisciplinary approach. In the present analysis, both fossil and biogeographic informations are considered in a qualitative manner to support hypothetical colonization processes.

## Results

### Phylogenetic and phylogeographic analyses

The phylogeographic structure of the common vole in Europe was investigated from cytb (1044 bp) and CR (304 bp) fragments (Figures 1B–1C and Tables S1, S2). These fragments were not concatenated for phylogenetic reconstructions because they concern different specimens. Instead, the CR dataset (French populations) was analyzed mainly for correlation with the cytb dataset (European populations) on the genetic structure of the lineages potentially involved in postglacial colonization. The cytb sequences specified 209 variable sites defining 116 haplotypes (Table S3), and 113 of these polymorphic sites were phylogenetically informative (CR = 141 variable and 105 informative sites).

**Figure 2:**
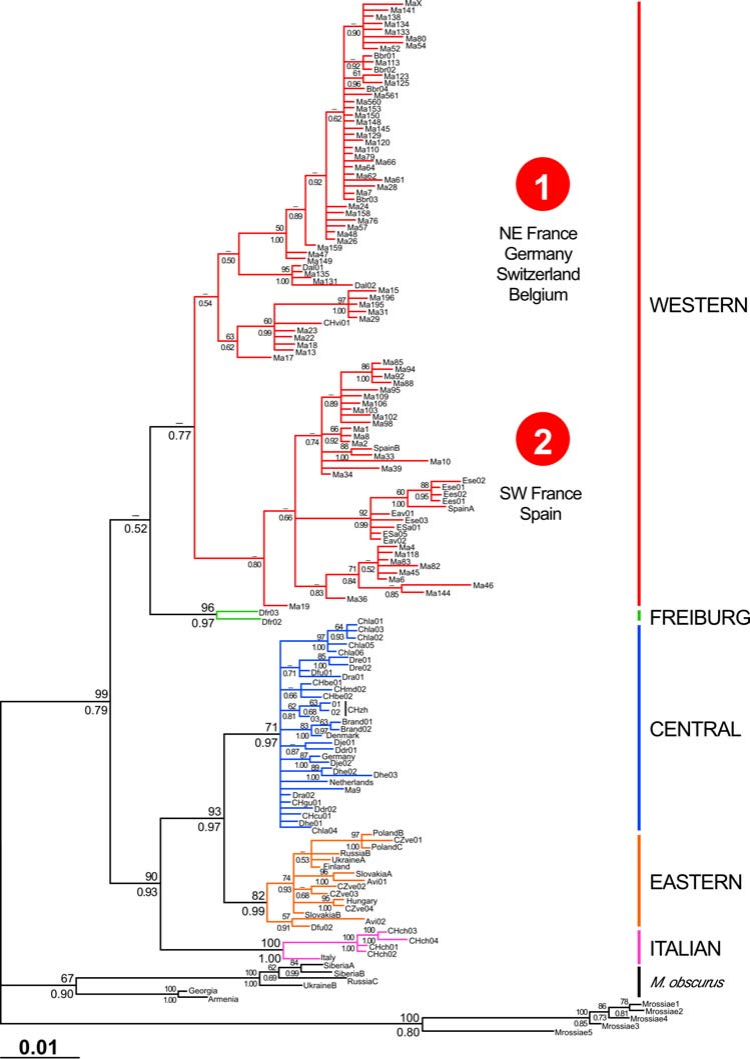
Bayesian tree reconstructed from cytochrome *b* gene sequences of *Microtus arvalis*. Individual labels are detailed in Table S1. The numbers at nodes refer to ML bootstrap percentages (above branches) and BA posterior probabilities (below branches). The five main evolutionary lineages as previously mentioned [34] are indicated on the right.

Phylogenetic analyses using the maximum-likelihood method (ML) and Bayesian approach (BA) provided congruent tree topologies for both cytb and CR dataset (Figure 2 and Figure S1). The cytb topology reflected the geographic origin of the specimens, and the five main evolutionary lineages of Fink *et al.* [33] and Heckel *et al.* [34] were found: W (France, Germany, Switzerland, Belgium, Spain), F (Germany), I (Switzerland, Italy), E (Germany, Austria, Hungary, Slovakia, Czech Republic, Poland, Ukraine, Russia, Finland) and C (France, Germany, Switzerland, Netherlands, Denmark). A high consistency was observed for the C, E, and I lineages with previously published analyses, whereas the W and F lineages seemed more closely related. However, the WF cluster and the W lineage were the least supported clades, with ML bootstrap percentages (BP) lower than 50%, and BA posterior probabilities (PP) between 0.50 and 0.80. Alternative hypotheses were investigated using the Shimodaira & Hasegawa test [39]. Over the 105 possible tree topologies among the five lineages, 34 trees were not significantly worse than the highest likelihood tree at the 5% confidence level (0.46<*P*<1; Table S4). The best ML tree placed the C lineage as the first ramification of the *M. arvalis* lineages, and the I and E lineages as the sister groups of the WF and WFI clusters, respectively. As such, it differed from the tree shown in Figure 2 (*P* = 0.976) and previously published trees (*P* = 0.993) [33], [34]. These topology differences can be explained by the low number of sequences clustered in the F and I lineages (respectively, 2 and 5). Phylogenetic analyses displayed a substructure within the W lineage (cytb: BP<50%; 0.54<PP<0.80; CR: 81%<BP<85%; PP = 0.87): (i) a northeastern (NE) group with specimens from NE France, Belgium, Germany and Switzerland; and (ii) a southwestern (SW) group with specimens from SW France and Spain. The Spanish common voles formed a monophyletic clade (92% BP, and 0.99 PP) that appeared to be the last offshoot of the SW group. This hierarchical phylogeographic structure of the W lineage had partly been revealed in previously published analyses with a smaller dataset [33], [34]. Conversely, the C and E lineages had cytb star-like topologies (Figure 2).

**Figure 3:**
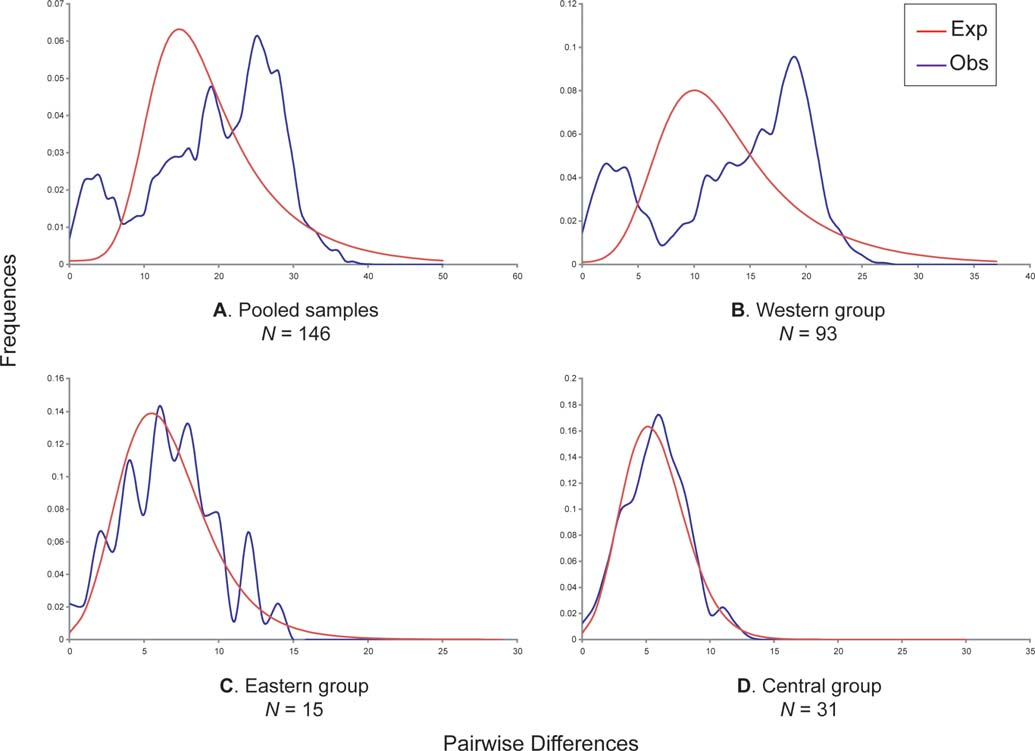
Demographic history of *Microtus arvalis* inferred from cytochrome *b* gene dataset. Observed mismatch distributions (blue line) for the whole dataset (A) as well as the Western (B), Eastern (C) and Central (D) lineages are compared to expected distributions under a population growth-decline model (red line). Numbers of pairwise differences are on the *X*-axis, while relative frequencies are on the *Y*-axis.

Nucleotide and haplotype diversities, as well as total and net DNA divergences were calculated for each lineage (Table 1). The W lineage had the highest nucleotide diversity (1.31%), while the I lineage had the lowest (0.49%). Nucleotide diversity for the other lineages was between 0.53% and 0.62%. The haplotype diversity was relatively high and homogeneous, from 0.90 (Italian) to 1 (Freiburg). Total and net DNA divergences between lineages were estimated at 1.44–3.18% and 0.86–2.40%, respectively. The I lineage showed the highest value. The nucleotide diversity and net DNA divergence were in agreement with previously published analyses [6], [33]. Demographic histories were inferred by a pairwise mismatch distribution analysis [40] (Figure 3). This analysis, when applied to the whole dataset, showed a heterogeneous distribution suggesting long-term stability. As pooling differentiated samples may induce bias, the mismatch distribution analyses were also performed for the W, C and E lineages separately. The W lineage presented a heterogeneous distribution; the C and E lineages had a bell-shaped distribution, suggesting a sudden expansion of these populations. As for alternative topology hypotheses, there were too few samples available for the F and I lineages (respectively, 2 and 5) to obtain reliable results.

**Table 1:** Genetic variability within and between *Microtus arvalis* lineages based on cytochrome *b* gene sequences.

Da^a^	Western		Eastern	Central	Freiburg	Italian	π^c^	*h* d
Dxy^b^	*NE*	*SW*						
Western	-	1.76 (0.12)	1.71 (0.08)	0.92 (0.23)	2.28 (0.25)	1.31 (0.05)	0.98	
*NE*	*-*	*0.90 (0.1)* e					*0.76 (0.08)*	*0.96*
*SW*	*1.80 (0.009)*	*-*					*1.02 (0.07)*	*0.99*
Eastern	2.72 (0.11)^f^	-	0.86 (0.11)	1.58 (0.50)	2.01 (0.42)	0.62 (0.09)	0.98	
Central	2.66 (0.08)	1.44 (0.11)	-	1.42 (0.34)	2.16 (0.31)	0.53 (0.05)	0.99	
Freiburg	1.87 (0.18)	2.18 (0.48)	1.99 (0.30)	-	2.40 (1.08)	0.58 (0.29)	1	
Italian	3.18 (0.22)	2.57 (0.40)	2.65 (0.29)	2.93 (1.07)	-	0.49 (0.21)	0.90	

a Net and ^b^total DNA divergences.

c Nucleotide and ^d^haplotype diversities.

e NE and SW values are in italics.

f Standard error.

### Divergence time estimates within and among *M. arvalis* lineages

**Figure 4:**
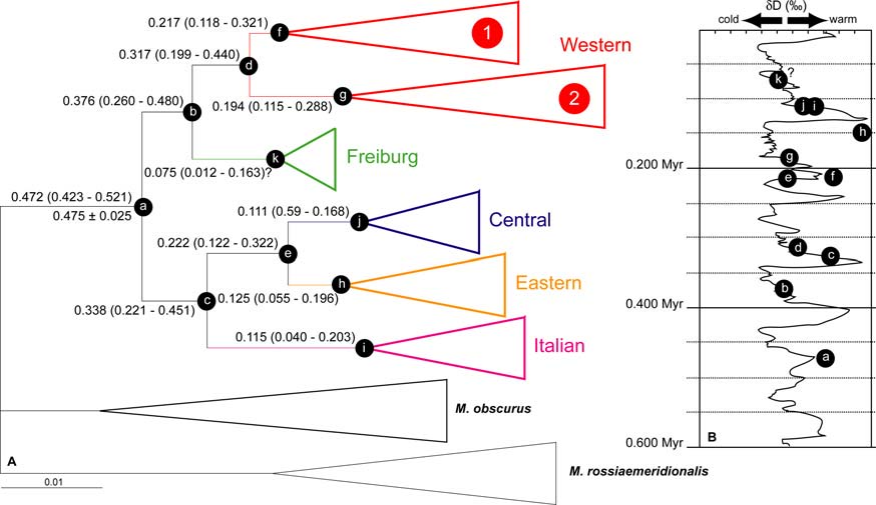
Divergence time estimates within and among *Microtus arvalis* lineages. Numbers at nodes (b to k) of the simplified Bayesian tree (A) are times to most recent common ancestors (with 95% confidence) estimated from cytochrome *b* gene sequences with BEAST. Numbers at node (a) are the divergence time estimate by BEAST (above branch) based on the fossil calibration point (below branch; Miesenheim I, Germany) [29], [30]. Small letters allow one to locate the possible lineage appearance on the δ*D* curve (B) from EPICA Dome C (modified from [75]).

As a consequence of the mismatch distribution analysis for the whole dataset, divergence dates of the main clades (Figure 4) were calculated under a Bayesian relaxed-clock method assuming constant population size. With the first occurrence of *M. arvalis* at 0.475±0.025 Myr (Miesenheim I, Germany) [29], [30], the mutation rate was estimated at 4.8 substitutions/site/Myr. The W lineage showed the oldest divergence time (0.317 Myr; 95% confidence 0.199–0.440 Myr), while the F lineage showed the most recent one (0.075 Myr; 95% confidence 0.012–0.163 Myr). However, this latter result should be considered with caution because the tree topology was not in agreement with such a recent divergence time. The two F sequences are probably insufficient for inferring reliable time estimates. As for the Spanish clade (data not shown), the most recent common ancestor was 0.086 Myr old (95% confidence 0.037–0.144 Myr).

The 95% confidence ranges of divergence time estimates are wide, making it difficult to correlate formation events of *M. arvalis* lineages to Quaternary climatic events. However, Bayesian posterior probability densities (data not shown) of these estimates are unimodal, indicating that mean estimates should provide a reasonable basis for inferences about the evolutionary history of *M. arvalis* [41].

The divergence time estimates among the five evolutionary lineages of *M. arvalis* covered several glacial and interglacial periods in the Middle and Late Pleistocene. This pattern is congruent with a pre-LGM origin, split and evolution of the lineages, although some previously published divergence times are much younger than the present molecular dating [33], [34]. Each of our estimates corresponds to warm or pre- and postglacial periods (Figure 4).

## Discussion

**Figure 5:**
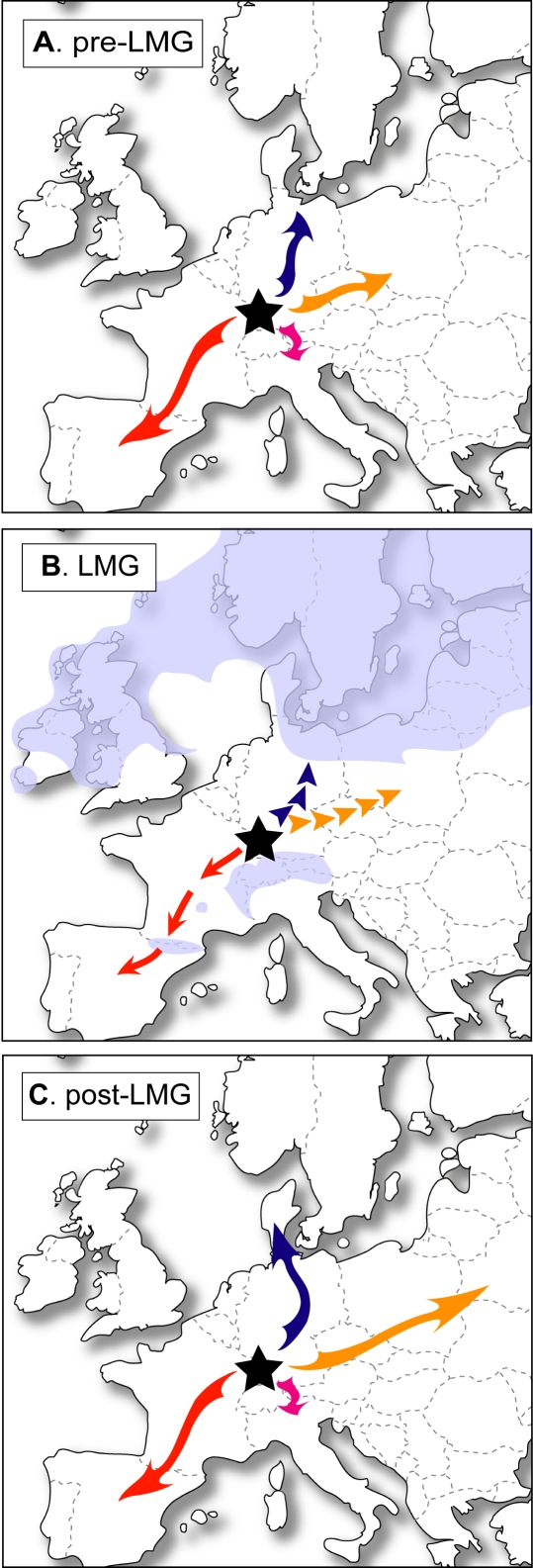
Estimated expansions of *Microtus arvalis* before (A), during (B) and after (C) the LGM. The black star locates the most likely origin of the species from fossil and genetic evidence. Solid arrows indicate the southwestward and northeastward gradual range expansion during warm periods, while dotted arrows are for more or less irregular expansion (Ù glacial survival in isolated populations or ä bottleneck events) during cold periods. Shaded areas (B) correspond to the estimated extension of ice cover.

The phylogeographic history of the common vole is characterized by a deep genetic differentiation of the five main evolutionary lineages (Western, Freiburg, Central, Eastern and Italian; Figures 2 and 4). Similarities with phylogeographic patterns of *M. arvalis* data from several genetic markers (cytb, CR, microsatellites) [33], [34], as well as data from other temperate species [3], [13], [42]–[45] suggest that the distribution of populations between Western and Eastern Europe reflects the evolutionary history of populations rather than genetic marker genealogy. Molecular dating indicates that the population divergence, from the lineage origin to the origin of the Spanish clade of *M. arvalis*, occurred during the Middle and Late Pleistocene (0.475–0.086 Myr), and thus predated the LGM widely. These molecular dating results coincide with warm or pre- and postglacial periods between the Late Cromerian and the LGM (Figure 4). The C and E lineages have a shallow regional genetic structure. Low nucleotide diversity (0.53% and 0.62%, respectively), star-like topologies (Figure 2) and analyses of demographic history indicating sudden expansion (Figure 3) provide evidence for a past bottleneck event followed by probable post-LGM population expansion [45]–[47]. The oldest major W lineage presents a higher level of nucleotide diversity (1.31%) suggestive of relatively large population sizes, and shows a hierarchical phylogeographic structure (NE and SW sublineages) as observed in the field vole [43]. However, these sublineages found on either side of the Loire River (France) also have different topological tree structures, reflecting different genetic structures (Table 1). The NE sublineage experienced to a lesser extent the effect of periglacial climatic conditions, while the SW populations located between the Atlantic and Mediterranean coasts were under milder climatic conditions (Figures 2, 5 and S1). The closer the populations were to the ice front, the more significant was the loss of genetic diversity.

On closer examination of lineage composition, individuals from Germany, France and Switzerland are found in nearly all lineages. German individuals belong to the W, F, C and E lineages, while individuals from Switzerland are from the W, C and I lineages. Particular attention should be paid to populations from eastern France, because some individuals from the Vittel and Monthureux populations belong not only to both W sublineages, but also to the C lineage (Figures 2 and S1) [33], [34]. Therefore, the *M. arvalis* lineages meet in an area including eastern France, southwestern Germany and northern Switzerland. Moreover, the fossil site of Miesenheim I, where the oldest *M. arvalis* remains were found (0.500–0.450 Myr) [29], [30], is located in southwestern Germany (Figure 1). For these reasons, we consider the origin of *M. arvalis* to be located in western Central Europe. From this area, populations of the common vole certainly spread out through Europe during the Holsteinian (0.430–0.300 Myr), first southwestwards and then northeastwards (Figure 5). Western Central Europe was not only the cradle of the *M. arvalis* lineages, it was also a dispersal centre for this rodent.

Mediterranean regions were long considered to have been refugia for numerous European temperate plants and animals during Pleistocene ice ages [3]–[5]. However, the role of these refugia for the survival of *M. arvalis* during the LGM has recently been challenged [34]. The Spanish clade has distinctive haplotypes (Table S3), and seems to be the last offshoot of the W lineage dated back 0.086 Myr (Figures 2 and 4). Likewise, the Italian lineage has distinctive haplotypes but also the highest DNA divergence (Tables 1 and S3). Even if the Spanish clade may result from a gradual expansion of the species from eastern France to Spain, both Spanish and Italian populations may represent long-term isolates, explaining their relatively high level of endemism [3], [8]. Therefore, these populations probably did not contribute to potential postglacial colonization of Europe. Central European glacial refugia (in the Carpathians), as opposed to Mediterranean refugia, have been proposed for some large mammals (*e.g.* brown bear, European badger, red deer) [16], [41], [48] and for several more small mammals adapted to open (root vole and field vole) [35], [43] and forest (bank vole and yellow-necked fieldmouse) [13], [44] landscapes. The fossil record indicates that *M. arvalis* was present in Mediterranean regions (Spain, Portugal, Italy, Greece) during both warm periods and glacials [30]. Surprisingly, this rodent has been recorded in fossil localities of Western (France), Central (Germany, Switzerland and Austria) and Eastern (Slovakia, Hungary, Romania) Europe from the Late Cromerian to the Holocene [30], regardless of the climatic conditions. Furthermore, the detailed examination of two French fossil localities, la Baume de Gigny (Gigny, Jura; 0.060–0.015 Myr) [49]–[51] and le Taillis des Coteaux (Antigny, Vienne; 0.030–0.015 Myr) [52] reveals that the common vole survived even in northern France during the LGM. We therefore suggest that glacial survival of *M. arvalis* could have occurred in patchy and restricted favourable habitats (as described by [11], [17]) on its whole geographic range rather than in southern or northern glacial refugia.

Biogeographic traits may have determined the responses of plant and animal species to Pleistocene ice ages [17]. When both fossil and genetic evidence for northerly glacial survival exists, the mammals concerned have short generation time and small body size (like voles), as well as high mobility. The common vole is not really a highly mobile rodent but it is able to travel distances of several hundred meters to a few kilometres [53]. This rodent has also shown ecological adaptation to cold environments. For example, it was reported at an altitude of 3000 m in the Alps, and it can survive several months, and even reproduce, under snow cover in mountainous areas [54]. However, landscape structure and composition as well as climatic conditions seem to be important constraints for species dispersal and population dynamics [55]–[57]. Open habitats and mean July temperatures >+16°C favour vole outbreak and the spread to all habitats around, while a mosaic landscape and arid environment prevent the dispersal of the species [25], [58], [59]. Analyses of climate dynamics and vegetation response during the past 0.140 Myr in Western and Central Europe allow the documentation of environmental changes. The Eemian interglacial (0.126–0.110 Myr) and the Weichselian (0.110–0.001 Myr) interstadials were characterized by closed forests or forest steppes and summer temperatures >+16°C. Landscapes of the Weichselian stadials were dominated by open vegetation typical of tundra, steppe tundra, tundra woodland or possibly open taiga, and mean July temperatures were ≤10°C [37], [60]–[62]. Our molecular dating results indicate that the dispersal of *M. arvalis* was effective during warm, pre- and postglacial periods, and not in cold periods (Figure 4). Warm climatic conditions were indeed more suitable for this species, and open habitats (floodplains, open woodlands, scrub, woodland glades, open marshes and meadows) were sufficiently well represented to allow for its existence and expansion [63]. Moreover, the high haplotype diversity (Table 1) suggests glacial survival of small, isolated populations in microenvironmentally favourable habitats from which local dispersal was then possible [11], [17]. A relationship between the ratio of permanent grassland and the kinetics of *M. arvalis* was also brought to light. When grassland represents less than 10% of cultivated environments, *M. arvalis* densities remain constantly low in those grassland habitats. However, when grassland is the main constituent of these environments transformed by human activity, *M. arvalis* populations may present episodes of high densities (hundred individuals/ha) [59]. The common vole seems therefore able to survive harsh environmental conditions (*i.e.* snow cover, patchy habitats). The species can then disperse from grassland patch to patch by increasing population density during more suitable conditions. This capacity could therefore allow the expansion of this species during transitional phases such as pre- and postglacial periods (relatively open landscape and mild climate conditions).

Therefore, combined fossil, genetic and biogeographic evidence provides a new insight into the evolutionary history of *M. arvalis*. Different colonization processes from western Central Europe allowed the dispersal of this species from small isolated populations rather than from southern or northern glacial refugia. These colonization directions included: (i) southwestwards, with a relatively gradual range expansion during suitable periods in Western Europe; and (ii) northeastwards, with a more irregular expansion in Central and Eastern Europe. In this latter case, populations might not retreat towards glacial refugia, only to perish because of periglacial conditions. The lack of fossil remains in Denmark, Poland, Russia and Ukraine for the LGM [30] and the genetic evidence of a past C and E bottleneck event strengthen these hypotheses. In fact, the dispersal of the common vole throughout Europe is due to a balance between favourable structure and composition of the landscape and climatic conditions. Thus, the evolutionary history of European temperate species is probably the result of much more complex colonization processes than usually thought. Fossil and genetic evidence as well as biogeographic traits should lead to the improved management and conservation of modern biodiversity.

## Materials and Methods

### Samples

Voles were euthanized by cervical dislocation as recommended by our institutions (http://ethique.ipbs.fr/sdv/GUIDEmars2008.pdf) and Mills *et al.* [64]. One of the authors (JPQ) has an authorization to experiment on living vertebrate animals (Certificate n° 34.107). Phylogeographic inferences are based on agreement in analysis of 131 control region and 75 cytochrome *b* gene sequences from common voles, sampled from 35 French localities (1 to 9 individuals/population; Figure 1 and Tables S1, S2). Our molecular dataset was complemented with GenBank sequences including *M. arvalis* from other European localities and *M. rossiaemeridionalis* used as outgroup (Figure 1 and Tables S1, S2).

From a palaeontological standpoint, the whole European fossil record of *M. arvalis* was considered [30]. Kowalski [30] listed *M. arvalis* remains in two categories: *M. arvalis*/*M. agrestis* assemblages and *M. arvalis* localities. In the present study, only the second category with well-determined specimens is taken into account. Two continuous and well-studied sequences located in France were also examined (Figure 1): La Baume de Gigny (Gigny, Jura; 0.060–0.015 Myr) [49]–[51] and le Taillis des Coteaux (Antigny, Vienne; 0.030–0.015 Myr) [52]. In the former locality, *M. arvalis* and *M. agrestis* were identified [49], while *M. arvalis* remains of the latter locality were determined by one of us (ER in [52]).

### DNA extraction, amplification and sequencing

Total DNA was extracted from 95% ethanol-preserved liver, foot and skin fragments following standard procedures [65]. Because of their relatively fast substitution rate, the control region and the cytochrome *b* gene are frequently used as genetic marker in phylogeographic studies dealing with, vertebrates in general, and mammals in particular [*e.g.* 3], [6], [7], [10], [13], [15], [18], [20], [23], [34]. For this reason, the CR 5′ peripheral domain (more informative than the 3′) and the cytb gene were PCR-amplified (Tm = 50°C) with, respectively, specific and universal primers (Table S5) [28], [66], [67]. Direct sequencing was performed in both directions to confirm polymorphic sites by Macrogen (Seoul, Korea). The new sequences were deposited in the EMBL Nucleotide Sequence Database under accession numbers AM990179–AM990312 for CR, and AM991024–AM991098 for cytb (see Table S1 for sequence details).

### Data analysis

Best-fitting models of sequence evolution were determined using Modeltest 3.7 [68] for ML reconstructions and MrModeltest 2.2 [69] for BA. These models are: K81uf+I+G (ML) and GTR+I+G (BA) for CR; TrN+I+G (ML) and K80+I, GTR, GTR+G (respectively, first, second and third codon positions; BA) for cytb (for details about models, see http://workshop.molecularevolution.org/eur/resources/models/dnamodels.php). ML analyses were conducted using the software PhyML 2.4.4 [70], and nodal robustness was estimated after 1000 bootstrap replicates. Alternative topologies to the best ML tree were evaluated with the test of Shimodaira & Hasegawa [39] as implemented in PAUP* 4.010b [71]. Mixed models under BA using MrBayes 3.0b4 [72] was performed with five Markov chain Monte Carlo chains that were simultaneously run for 2,000,000 generations with trees sampled every 100^th^ generation, and after removing the first 2000 trees as the burn-in stage.

Nucleotide and haplotype diversities within evolutionary lineages, as well as total and net DNA divergence were calculated using DnaSP 4.20.2 [73]. Mismatch distribution analyses among individuals [40] were carried out under a population growth-decline model in DnaSP. Demographic stability is illustrated by multimodal distributions, while a unimodal pattern is consistent with sudden expansion [46].

### Divergence time estimates

Time to most recent common ancestor for several clades was estimated from cytb dataset using BA with BEAST 1.4.6 [74] under a GTR+I+G model. Runs were performed with an uncorrelated lognormal clock assuming constant population size (20,000,000 generations with the first 2,000,000 discarded as burn-in). The date of 0.475±0.025 Myr for the origin of *M. arvalis* lineages (Late Cromerian; Miesenheim I, Germany) [29], [30] was used as calibration point.

## Supporting Information

Table S1 Labels, geographic distribution and references/sources of Microtus arvalis samples. Accession numbers for original and Genbank data of the cytochrome b gene and the control region are also listed. Colours refer to the five lineages: Western (red), Central (blue), Eastern (orange), Freiburg (green) and Italian (pink).
(0.34 MB DOC)

Table S2 Origin and sample size of Microtus arvalis
(0.13 MB DOC)

Table S3 Haplotype list for each Microtus arvalis lineage. The positions of changes in the amino acid sequence are given according to the cytochrome b model by Howell [79] and Degli Esposti et al. [80].
(0.17 MB DOC)

Table S4 Alternative topologies nonsignificantly different (5% confidence level) including the five lineages of Microtus arvalis. The topology in bold is the previously published topology [33], [34], while the topology in red corresponds to the present topology based on cytochrome b gene sequences (Figure 2).
(0.09 MB DOC)

Table S5 Primers used for PCR-amplification of the cytochrome b gene [28], [67] and the control region [66]
(0.03 MB DOC)

Figure S1 Bayesian tree reconstructed from control region sequences of Microtus arvalis. Individual labels are detailed in Table S1. The numbers at nodes refer to ML bootstrap percentages ≥50% (above branches) and BA posterior probabilities ≥0.50 (below branches). The five main evolutionary lineages as previously mentioned [34] are indicated on the right.
(0.34 MB DOC)
