# Supplementary material for: New Insight into the Colonization Processes of Common Voles: Inferences from Molecular and Fossil Evidence
Source: PLoS One. 2008 Oct 29;3(10):e3532. doi: 10.1371/journal.pone.0003532 (PMC2570793; doi:10.1371/journal.pone.0003532)
Supplement: Table S1 — Labels, geographic distribution and references/sources of Microtus arvalis samples. Accession numbers for original and Genbank data of the cytochrome b gene and the control region are also listed. Colours refer to the five lineages: Western (red), Central (blue), Eastern (orange), Freiburg (green) and Italian (pink). (0.34 MB DOC) [file pone.0003532.s001.doc]

**Table S1.** Labels, geographic distribution and references/tissue sources of *Microtus arvalis* samples.

Accession numbers for original and GenBank data of the cytochrome *b* gene and control region are also listed. Colours refer to the five lineages: Western (red), Central (blue), Eastern (orange), Freiburg (green) and Italian (pink).

| Label | Location | Accession number | | References/Tissue sources |
| --- | --- | --- | --- | --- |
|  |  | Control region | Cytochrome *b* |  |
| **France** |  |  |  |  |
| **Ma1** | Armendarits (64)^a^ | AM990179 | AM991024 | JPQ |
| **Ma2** | Hasparren (64) | AM990180 | AM991025 | JPQ |
| **Ma4** | Pompertuzat (31) | AM990181 | AM991026 | JPQ |
| **Ma5** | Pompertuzat (31) | AM990182 | - | JPQ |
| **Ma6** | Pompertuzat (31) | AM990183 | AM991027 | JPQ |
| **Ma7** | Monthureux-le-Sec (88) | AM990184 | AM991028 | JPQ |
| **Ma8** | Monthureux-le-Sec (88) | AM990185 | AM991029 | JPQ |
| **Ma9** | Monthureux-le-Sec (88) | AM990186 | AM991030 | JPQ |
| **Ma10** | Espézel (11) | AM990187 | AM991031 | JPQ |
| **Ma11** | Espézel (11) | AM990188 | - | JPQ |
| **Ma12** | Espézel (11) | AM990189 | - | JPQ |
| **Ma13** | Vanoise National Parc (73) | AM990190 | AM991032 | M. Bouche |
| **Ma14** | Vanoise National Parc (73) | AM990191 | - | M. Bouche |
| **Ma15** | Vanoise National Parc (73) | AM990192 | AM991033 | M. Bouche |
| **Ma17** | Monétier-les-Bains (05) | AM990193 | AM991034 | JPQ |
| **Ma18** | Monétier-les-Bains (05) | AM990194 | AM991035 | JPQ |
| **Ma19** | Espézel (11) | AM990195 | AM991036 | JPQ |
| **Ma20** | Monétier-les-Bains (05) | AM990196 | - | JPQ |
| **Ma21** | Monétier-les-Bains (05) | AM990197 | - | JPQ |
| **Ma22** | Monétier-les-Bains (05) | AM990198 | AM991037 | JPQ |
| **Ma23** | Monétier-les-Bains (05) | AM990199 | AM991038 | JPQ |
| **Ma24** | Vittel (88) | - | AM991039 | JPQ |
| **Ma25** | Vittel (88) | AM990200 | - | JPQ |
| **Ma26** | Vittel (88) | AM990201 | AM991040 | JPQ |
| **Ma27** | Vittel (88) | AM990202 | - | JPQ |
| **Ma28** | Vittel (88) | AM990203 | AM991041 | JPQ |
| **Ma29** | Vanoise National Parc (73) | AM990204 | AM991042 | M. Bouche |
| **Ma30** | Vanoise National Parc (73) | AM990205 | - | M. Bouche |
| **Ma31** | Vanoise National Parc (73) | AM990206 | AM991043 | M. Bouche |
| **Ma33** | Espézel (11) | - | AM991044 | JPQ |
| **Ma34** | Néouvielle Natural Reserve (65) | AM990207 | AM991045 | JPQ/CT |
| **Ma35** | Néouvielle Natural Reserve (65) | AM990208 | - | JPQ/CT |
| **Ma36** | Néouvielle Natural Reserve (65) | AM990209 | AM991046 | JPQ/CT |
| **Ma37** | Néouvielle Natural Reserve (65) | AM990210 | - | JPQ/CT |
| **Ma38** | Néouvielle Natural Reserve (65) | AM990211 | - | JPQ/CT |
| **Ma39** | Néouvielle Natural Reserve (65) | AM990212 | AM991047 | JPQ/CT |
| **Ma41** | Monétier-les-Bains (05) | AM990213 | - | JPQ |
| **Ma43** | Fourchambault (53) | AM990214 | - | D. Sirugue |
| **Ma44** | Armendarits (64) | AM990215 | - | JPQ |
| Label | Location | Accession number | | References/Tissue sources |
|  |  | Control region | Cytochrome *b* |  |
| **Ma45** | La Force (24) | AM990216 | AM991048 | E. Fichet-Calvet |
| **Ma46** | La Force (24) | AM990217 | AM991049 | E. Fichet-Calvet |
| **Ma47** | Septfontaines (25) | AM990218 | AM991050 | D. Michelat |
| **Ma48** | Septfontaines (25) | AM990219 | AM991051 | D. Michelat |
| **Ma49** | Septfontaines (25) | AM990220 | - | D. Michelat |
| **Ma51** | Chapelle d'Huin (25) | AM990221 | - | D. Michelat |
| **Ma52** | Chapelle d'Huin (25) | AM990222 | AM991052 | D. Michelat |
| **Ma54** | Chapelle d'Huin (25) | AM990223 | AM991053 | D. Michelat |
| **Ma55** | Chapelle d'Huin (25) | AM990224 | - | D. Michelat |
| **Ma57** | Pont du Château (63) | AM990225 | AM991054 | B. Pradier |
| **Ma58** | Pont du Château (63) | AM990226 | - | B. Pradier |
| **Ma59** | Pont du Château (63) | AM990227 | - | B. Pradier |
| **Ma60** | Pont du Château (63) | AM990228 | - | B. Pradier |
| **Ma61** | Pont du Château (63) | AM990229 | AM991055 | B. Pradier |
| **Ma62** | Monnaie (37) | AM990230 | AM991056 | J.-P. Damange |
| **Ma63** | Monnaie (37) | AM990231 | - | J.-P. Damange |
| **Ma64** | Monnaie (37) | - | AM991057 | J.-P. Damange |
| **Ma65** | Monnaie (37) | AM990232 | - | J.-P. Damange |
| **Ma66** | Monnaie (37) | AM990233 | AM991058 | J.-P. Damange |
| **Ma76** | Abbéville-la-Rivière (91) | AM990234 | AM991059 | G. Grolleau |
| **Ma77** | Abbéville-la-Rivière (91) | AM990235 | - | G. Grolleau |
| **Ma78** | Abbéville-la-Rivière (91) | AM990236 | - | G. Grolleau |
| **Ma79** | Abbéville-la-Rivière (91) | AM990237 | AM991060 | G. Grolleau |
| **Ma80** | Brion (15) | AM990238 | AM991061 | JPQ |
| **Ma81** | Brion (15) | AM990239 | - | JPQ |
| **Ma82** | Brion (15) | AM990240 | AM991062 | JPQ |
| **Ma83** | Brion (15) | AM990241 | AM991063 | JPQ |
| **Ma84** | Brion (15) | AM990242 | - | JPQ |
| **Ma85** | Noirmoutiers (85) | AM990243 | AM991064 | JPQ |
| **Ma86** | Noirmoutiers (85) | AM990244 | - | JPQ |
| **Ma87** | Noirmoutiers (85) | AM990245 | - | JPQ |
| **Ma88** | Noirmoutiers (85) | AM990246 | AM991065 | JPQ |
| **Ma89** | Noirmoutiers (85) | AM990247 | - | JPQ |
| **Ma90** | Noirmoutiers (85) | AM990248 | - | JPQ |
| **Ma92** | Noirmoutiers (85) | AM990249 | AM991066 | JPQ |
| **Ma93** | Noirmoutiers (85) | AM990250 | - | JPQ |
| **Ma94** | Noirmoutiers (85) | AM990251 | AM991067 | JPQ |
| **Ma95** | Férel (56) | AM990252 | AM991068 | JPQ |
| **Ma97** | Férel (56) | AM990253 | - | JPQ |
| **Ma98** | Férel (56) | AM990254 | AM991069 | JPQ |
| **Ma99** | Férel (56) | AM990255 | - | JPQ |
| **Ma100** | Lusignan (86) | AM990256 | - | JPQ |
| **Ma101** | Lusignan (86) | AM990257 | - | JPQ |
| **Ma102** | Lusignan (86) | AM990258 | AM991070 | JPQ |
| **Ma103** | Lusignan (86) | AM990259 | AM991071 | JPQ |
| **Ma104** | Lusignan (86) | AM990260 | - | JPQ |
| **Ma105** | Saint Michel en l'Herm (85) | AM990261 | - | JPQ |
| Label | Location | Accession number | | References/Tissue sources |
|  |  | Control Region | Cytochrome *b* |  |
| **Ma106** | Saint Michel en l'Herm (85) | AM990262 | AM991072 | JPQ |
| **Ma108** | Saint Michel en l'Herm (85) | AM990263 | - | JPQ |
| **Ma109** | Saint Michel en l'Herm (85) | AM990264 | AM991073 | JPQ |
| **Ma110** | Le Neubourg (27) | AM990265 | AM991074 | CT |
| **Ma112** | Evreux (27) | AM990266 | - | Y. Tougard |
| **Ma113** | Evreux (27) | AM990267 | AM991075 | Y. Tougard |
| **Ma114** | Evreux (27) | AM990268 | - | Y. Tougard |
| **Ma118** | Les Rives (34) | AM990269 | AM991076 | JPQ |
| **Ma119** | Les Rives (34) | AM990270 | - | JPQ |
| **Ma120** | Coublanc (71) | AM990271 | AM991077 | CT |
| **Ma123** | Thise (25) | AM990272 | AM991078 | D. Truchetet |
| **Ma124** | Thise (25) | AM990273 | - | D. Truchetet |
| **Ma125** | Thise (25) | AM990274 | AM991079 | D. Truchetet |
| **Ma126** | Thise (25) | AM990275 | - | D. Truchetet |
| **Ma127** | Thise (25) | AM990276 | - | D. Truchetet |
| **Ma129** | Thise (25) | AM990277 | AM991080 | D. Truchetet |
| **Ma130** | Wiwersheim (67) | AM990278 | - | J. Gaspar |
| **Ma131** | Wiwersheim (67) | AM990279 | AM991081 | J. Gaspar |
| **Ma132** | Wiwersheim (67) | AM990280 | - | J. Gaspar |
| **Ma133** | Wiwersheim (67) | AM990281 | AM991082 | J. Gaspar |
| **Ma134** | Otterswiller (67) | AM990282 | AM991083 | J. Gaspar |
| **Ma135** | Otterswiller (67) | AM990283 | AM991084 | J. Gaspar |
| **Ma136** | Otterswiller (67) | AM990284 | - | J. Gaspar |
| **Ma137** | Otterswiller (67) | AM990285 | - | J. Gaspar |
| **Ma138** | Otterswiller (67) | AM990286 | AM991085 | J. Gaspar |
| **Ma139** | Otterswiller (67) | AM990287 | - | J. Gaspar |
| **Ma140** | Otterswiller (67) | AM990288 | - | J. Gaspar |
| **Ma141** | Otterswiller (67) | AM990289 | AM991086 | J. Gaspar |
| **Ma142** | Les Rives (34) | AM990290 | - | JPQ |
| **Ma143** | Les Rives (34) | AM990291 | - | JPQ |
| **Ma144** | Les Rives (34) | AM990292 | AM991087 | JPQ |
| **Ma145** | Chateaudouble (26) | AM990293 | AM991088 | JPQ |
| **Ma146** | Chateaudouble (26) | AM990294 | - | JPQ |
| **Ma147** | Chateaudouble (26) | AM990295 | - | JPQ |
| **Ma148** | Chateaudouble (26) | AM990296 | AM991089 | JPQ |
| **Ma149** | Chateaudouble (26) | AM990297 | AM991090 | JPQ |
| **Ma150** | Nuit-Saint-Georges (21) | AM990298 | AM991091 | C. Thomas |
| **Ma151** | Nuit-Saint-Georges (21) | AM990299 | - | C. Thomas |
| **Ma153** | Nuit-Saint-Georges (21) | AM990300 | AM991092 | C. Thomas |
| **Ma154** | Nuit-Saint-Georges (21) | AM990301 | - | C. Thomas |
| **Ma155** | Nuit-Saint-Georges (21) | AM990302 | - | C. Thomas |
| **Ma158** | Vauville (50) | AM990303 | AM991093 | A. Labouille |
| **Ma159** | Vauville (50) | AM990304 | AM991094 | A. Labouille |
| **Ma195** | La Clusaz (74) | AM990305 | AM991095 | P. Brunet-Lecomte |
| **Ma196** | La Clusaz (74) | AM990306 | AM991096 | P. Brunet-Lecomte |
| **Ma560** | Calais (62) | AM990307 | AM991097 | J. B. Searle |
| **Ma561** | Calais (62) | AM990308 | AM991098 | J. B. Searle |
| Label | Location | Accession number | | References/Tissue sources |
|  |  | Control region | Cytochrome *b* |  |
| **Ma562** | Calais (62) | AM990309 | - | J. B. Searle |
| **Spain B** | Mantet (66) | - | AY220789 | [6] |
| **Spain** |  |  |  |  |
| **Eav01** | Avila | - | AY708499 | [33] |
| **Eav02** | Avila | - | AY708500 | [33] |
| **Ees01** | El Espinar | - | AY708523 | [33] |
| **Ees02** | El Espinar | - | AY708524 | [33] |
| **Esa01** | Salamanca | - | AY708502 | [33] |
| **Esa05** | Salamanca | - | AY708501 | [33] |
| **Ese01** | Segovia | - | AY708525 | [33] |
| **Ese02** | Segovia | - | AY708497 | [33] |
| **Ese03** | Segovia | - | AY708503 | [33] |
| **Spain A** | Fuentes de Nava | - | AY220788 | [6] |
| **Belgium** |  |  |  |  |
| **Bbr01** | Brussels | - | AY708508 | [33] |
| **Bbr02** | Brussels | - | AY708509 | [33] |
| **Bbr03** | Brussels | - | AY708510 | [33] |
| **Bbr04** | Brussels | - | AY708462 | [33] |
| **Austria** |  |  |  |  |
| **Avi01** | Vienna | - | AY708460 | [33] |
| **Avi02** | Vienna | - | AY708461 | [33] |
| **Arv1** | Zeiselmauer | AF267285 | - | [66] |
| **Switzerland** |  |  |  |  |
| **CHbe01** | Belp | - | AY708463 | [33] |
| **CHch01** | Chur | - | AY708512 | [33] |
| **CHch02** | Chur | - | AY708513 | [33] |
| **CHch03** | Chur | - | AY708465 | [33] |
| **CHch04** | Chur | - | AY708466 | [33] |
| **CHcu01** | Cudrefin | - | AY708482 | [33] |
| **CHgu01** | Gurnigel | - | AY708519 | [33] |
| **CHla01** | Lausanne | - | AY708514 | [33] |
| **CHla02** | Lausanne | - | AY708515 | [33] |
| **CHla03** | Lausanne | - | AY708516 | [33] |
| **CHla04** | Lausanne | - | AY708467 | [33] |
| **CHla05** | Lausanne | - | AY708468 | [33] |
| **CHla06** | Lausanne | - | AY708469 | [33] |
| **CHmd02** | Mühledorf | - | AY708486 | [33] |
| **CHvi01** | Visp | - | AY708481 | [33] |
| **CHzh01** | Zürich | - | AY708517 | [33] |
| **CHzh02** | Zürich | - | AY708518 | [33] |
| **CHzh03** | Zürich | - | AY708470 | [33] |
| **Italy** |  |  |  |  |
| **Italy** | Trento | - | AY220766 | [6] |
| **Germany** |  |  |  |  |
| **Dal01** | Alflen | - | AY708489 | [33] |
| **Dal02** | Alflen | - | AY708490 | [33] |
| **Ddr01** | Dresden | - | AY708491 | [33] |
| Label | Location | Accession number | | References/Tissue sources |
|  |  | Control region | Cytochrome *b* |  |
| **Ddr02** | Dresden | - | AY708498 | [33] |
| **Dfr02** | Freiburg | - | AY708493 | [33] |
| **Dfr03** | Freiburg | - | AY708507 | [33] |
| **Dfu01** | Furth im Wald | - | AY708474 | [33] |
| **Dfu02** | Furth im Wald | - | AY708475 | [33] |
| **Dhe01** | Heilsbronn | - | AY708476 | [33] |
| **Dhe02** | Heilsbronn | - | AY708477 | [33] |
| **Dhe03** | Heilsbronn | - | AY708478 | [33] |
| **Dje01** | Jena | - | AY708479 | [33] |
| **Dje02** | Jena | - | AY708480 | [33] |
| **Dra01** | Rastatt | - | AY708494 | [33] |
| **Dra02** | Rastatt | - | AY708520 | [33] |
| **Dre01** | Regensburg | - | AY708495 | [33] |
| **Dre02** | Regensburg | - | AY708496 | [33] |
| **Brand01** | Brandenburg | - | DQ768133 | Essbauer et *al.* (unpublished) |
| **Brand02** | Brandenburg | - | DQ768136 | Essbauer et *al.* (unpublished) |
| **Germany** | Göttingen | - | AY220777 | [6] |
| **Hungary** |  |  |  |  |
| **Hungary** | Nagycsány | - | AY220769 | [6] |
| **Netherlands** |  |  |  |  |
| **Netherlands** | Lauwersee | - | AY220778 | [6] |
| **Denmark** |  |  |  |  |
| **Denmark** | Hjerl Hede | - | AY220776 | [6] |
| **Finland** |  |  |  |  |
| **Finland** | Nuijamaa | - | AY220770 | [6] |
| **Slovakia** |  |  |  |  |
| **Slovakia A** | Velké Kosihy | - | AY220767 | [6] |
| **Slovakia B** | Stebník | - | AY220768 | [6] |
| **Czech Republic** |  |  |  |  |
| **CZve01** | Vetrkovice | - | AY708471 | [33] |
| **CZve02** | Vetrkovice | - | AY708472 | [33] |
| **CZve03** | Vetrkovice | - | AY708473 | [33] |
| **CZve04** | Vetrkovice | - | AY708505 | [33] |
| **Poland** |  |  |  |  |
| **Poland B** | Polkowo | - | AY220772 | [6] |
| **Poland C** | Kapice | - | AY220774 | [6] |
| **Ukraine** |  |  |  |  |
| **Ukraine A** | Chernobyl | - | U54488 | [76] |
| **Russia** |  |  |  |  |
| **Russia B** | Vladimir | - | AY220771 | [6] |
| **Unknown** |  |  |  |  |
| **MaX** | - | - | AF159403 | [77] |
| **MaY** | - | AJ009883 | - | Stacy & Ehrich (unpublished) |
| Label | Location | Accession number | | References/Tissue  sources |
|  |  | Control region | Cytochrome *b* |  |
| ***obscurus*** |  |  |  |  |
| Georgia | Ninotsminda | - | AY220760 | [6] |
| Armenia | Sisian | - | AY220761 | [6] |
| Ukraine B | Crimea | - | AY220762 | [6] |
| Russia C | Dyakov Forest | - | AY220763 | [6] |
| Siberia A | Kavka River | - | AY220764 | [6] |
| Siberia B | Neiva River | - | AY220765 | [6] |
| Mo4 | Baihaba, Xinjiang, China | AM990310 | - | JPQ |
| Mo9 | Baihaba, Xinjiang, China | AM990311 | - | JPQ |
| Mo16 | Narati, Xinjiang, China | AM990312 | - | JPQ |
| **Outgroup** |  |  |  |  |
| Mrossiae1 | Chernobyl | - | U54495 | [76] |
| Mrossiae2 | - | DQ015676 | DQ015676 | [26] |
| Mrossiae3 | Kauhava, Finland | - | AY513819 | [78] |
| Mrossiae4 | Svalbard, Norway | - | AY513820 | [78] |
| Mrossiae5 | Gerede, Turkey | - | AY513821 | [78] |
| Ma/Mr | Novosibirsk, Russia | DQ121418 | - | Yashina et *al.* (unpublished) |

^a^First two numbers of French zip codes
