# Supplementary material for: New Insight into the Colonization Processes of Common Voles: Inferences from Molecular and Fossil Evidence
Source: PLoS One. 2008 Oct 29;3(10):e3532. doi: 10.1371/journal.pone.0003532 (PMC2570793; doi:10.1371/journal.pone.0003532)
Supplement: Table S2 — Origin and sample size of Microtus arvalis (0.13 MB DOC) [file pone.0003532.s002.doc]

**Table S2.** Origin and sample size of *Microtus arvalis*

| Origin | control region | cytochrome *b* gene | Origin | control region | cytochrome *b* gene |
| --- | --- | --- | --- | --- | --- |
| **France** |  |  | **Belgium** |  |  |
| Armendarits (64)^a^ | 2 | 1 | Brussels | - | 4 (G) |
| Hasparren (64) | 1 | 1 | **Italy** |  |  |
| Pompertuzat (31) | 3 | 2 | Trento | - | 1 (G) |
| Monthureux-le-Sec (88) | 3 | 3 | **Switzerland** |  |  |
| Vittel (88) | 4 | 3 | Belp | - | 1 (G) |
| Espézel (11) | 4 | 3 | Chur | - | 4 (G) |
| Vanoise National Parc (73) | 6 | 4 | Cudrefin | - | 1 (G) |
| Monétier-les-Bains (05) | 7 | 4 | Gurnigel | - | 1 (G) |
| Néouvielle Natural Reserve (65) | 6 | 3 | Lausanne | - | 6 (G) |
| Fourchambault (53) | 1 | - | Mühledorf | - | 1 (G) |
| La Force (24) | 2 | 2 | Zürich | - | 3 (G) |
| Septfontaines (25) | 3 | 2 | **Germany** |  |  |
| Chapelle d’Huin (25) | 4 | 2 | Alflen | - | 2 (G) |
| Thise (25) | 6 | 3 | Dresden | - | 2 (G) |
| Pont du Château (63) | 5 | 2 | Freiburg | - | 2 (G) |
| Monnaie (37) | 4 | 2 | Furth im Wald | - | 2 (G) |
| Abbéville-la-Rivière (91) | 4 | 2 | Heilsbronn | - | 3 (G) |
| Brion (15) | 5 | 3 | Jena | - | 2 (G) |
| Noirmoutiers (85) | 9 | 4 | Rastatt | - | 2 (G) |
| Saint Michel en l’Herm (85) | 4 | 4 | Regensburg | - | 2 (G) |
| Férel (56) | 4 | 2 | Brandenburg | - | 2 (G) |
| Lusignan (86) | 5 | 2 | Göttingen | - | 1 (G) |
| Le Neubourg (27) | 1 | 1 | **Hungary** |  |  |
| Evreux (27) | 3 | 1 | Nagycsány | - | 1 (G) |
| Les Rives (34) | 5 | 2 | **Netherlands** |  |  |
| Coublanc (71) | 1 | 1 | Lauwersee | - | 1 (G) |
| Wiwersheim (67) | 4 | 2 | **Denmark** |  |  |
| Otterswiller (67) | 8 | 4 | Hjerl Hede | - | 1 (G) |
| Chateaudouble (26) | 5 | 3 | **Finland** |  |  |
| Nuit-Saint-Georges (21) | 5 | 2 | Nuijamaa | - | 1 (G) |
| Vauville (50) | 2 | 2 | **Slovakia** |  |  |
| La Clusaz (74) | 2 | 2 | Velké Kosihy | - | 1 (G) |
| Callais (62) | 3 | 2 | Stebník | - | 1 (G) |
| Mantet (66) |  | 1 (G)^b^ | **Czech Republic** |  |  |
| **Spain** |  |  | Vetrkovice | - | 4 (G) |
| Avila | - | 2 (G) | **Poland** |  |  |
| El Espinar | - | 2 (G) | Polkowo | - | 1 (G) |
| Salamanca | - | 2 (G) | Kapice | - | 1 (G) |
| Segovia | - | 3 (G) | **Ukraine** |  |  |
| Fuentes de Nava | - | 1 (G) | Chernobyl | - | 1 (G) |
| **Austria** |  |  | Russia |  |  |
| Vienna | - | 2 (G) | Vladimir | - | 1 (G) |
| Zeiselmauer | 1 (G) | - | **Unknown** | 1 (G) | 1 (G) |

^a^First two numbers of French zip codes

^b^Data retrieved from GenBank
