# Supplementary material for: New Insight into the Colonization Processes of Common Voles: Inferences from Molecular and Fossil Evidence
Source: PLoS One. 2008 Oct 29;3(10):e3532. doi: 10.1371/journal.pone.0003532 (PMC2570793; doi:10.1371/journal.pone.0003532)
Supplement: Table S3 — Haplotype list for each Microtus arvalis lineage. The positions of changes in the amino acid sequence are given according to the cytochrome b model by Howell [79] and Degli Esposti et al. [80]. (0.17 MB DOC) [file pone.0003532.s003.doc]

**Table S3.** Haplotype list for each *Microtus arvalis* lineage

The positions of changes in the amino acid sequence are given according to the cytochrome *b* model by Howell [79] and Degli Esposti *et al*. [80].

| Lineages | Haplotypes | Samples | Amino acid positions |
| --- | --- | --- | --- |
| **WESTERN** | W1 | Ees01, Ees02, Ese01 |  |
|  | W2 | MaX |  |
|  | W3 | Spain A |  |
|  | W4 | Spain B |  |
|  | W5 | Chvi01 | 360 |
|  | W6 | Dal01, Ma135 |  |
|  | W7 | Dal02 | 32, 357 |
|  | W8 | Ese02 | 356 |
|  | W9 | Eav01 | 356 |
|  | W10 | Eav02 |  |
|  | W11 | Esa05 |  |
|  | W12 | Esa01 |  |
|  | W13 | Ese03 |  |
|  | W14 | Bbr01, Bbr02, Ma113 |  |
|  | W15 | Bbr03 |  |
|  | W16 | Bbr04, Ma7, Ma62,  Ma64, Ma79, Ma110,  Ma129, Ma148, Ma150,  Ma153, Ma560 |  |
|  | W17 | Ma1, Ma2, Ma8 |  |
|  | W18 | Ma4 |  |
|  | W19 | Ma6, Ma83 |  |
|  | W20 | Ma10 | 314, 333 |
|  | W21 | Ma13 | 198 |
|  | W22 | Ma15 | 37, 360 |
|  | W23 | Ma17 |  |
|  | W24 | Ma18 |  |
|  | W25 | Ma19 |  |
|  | W26 | Ma22 |  |
|  | W27 | Ma23 | 29 |
|  | W28 | Ma24, Ma26, Ma48 |  |
|  | W29 | Ma28 |  |
|  | W30 | Ma29, Ma195 | 360 |
|  | W31 | Ma31 | 29, 360 |
| Lineages | Haplotypes | Samples | Amino acid positions |
| **WESTERN** | W32 | Ma33 |  |
|  | W33 | Ma34 |  |
|  | W34 | Ma36 |  |
|  | W35 | Ma39 | 23 |
|  | W36 | Ma45 |  |
|  | W37 | Ma46 |  |
|  | W38 | Ma47 | 135 |
|  | W39 | Ma52 |  |
|  | W40 | Ma54 | 29, 135 |
|  | W41 | Ma57 |  |
|  | W42 | Ma61 |  |
|  | W43 | Ma66 | 37, 96 |
|  | W44 | Ma76 |  |
|  | W45 | Ma80 | 158 |
|  | W46 | Ma82 |  |
|  | W47 | Ma85, Ma92 |  |
|  | W48 | Ma88 | 29 |
|  | W49 | Ma94 | 43 |
|  | W50 | Ma95 | 102, 108 |
|  | W51 | Ma98 |  |
|  | W52 | Ma102 |  |
|  | W53 | Ma103 |  |
|  | W54 | Ma106 |  |
|  | W55 | Ma109 | 37 |
|  | W56 | Ma118 |  |
|  | W57 | Ma144 |  |
|  | W58 | Ma120 |  |
|  | W59 | Ma123 | 177, 238 |
|  | W60 | Ma125 | 238 |
|  | W61 | Ma131 |  |
|  | W62 | Ma133 |  |
|  | W63 | Ma134 | 82 |
|  | W64 | Ma138 |  |
|  | W65 | Ma141 |  |
|  | W66 | Ma145 | 190 |
|  | W67 | Ma149 |  |
|  | W68 | Ma158 |  |
|  | W69 | Ma159 |  |
|  | W70 | Ma196 | 360 |
| Lineages | Haplotypes | Samples | Amino acid positions |
| **WESTERN** | W71 | Ma561 | 60 |
| **FREIBURG** | F1 | Dfr02 |  |
|  | F2 | Dfr03 |  |
| **CENTRAL** | C1 | Denmark | 365 |
|  | C2 | Germany |  |
|  | C3 | Brand01, Brand02 | 365 |
|  | C4 | Netherlands | 215 |
|  | C5 | Chbe01 |  |
|  | C6 | Chbe02 | 309 |
|  | C7 | Chla04 |  |
|  | C8 | Chla05 |  |
|  | C9 | Chla06 |  |
|  | C10 | Chzh03 |  |
|  | C11 | Dfu01 |  |
|  | C12 | Dhe01 |  |
|  | C13 | Dhe02 | 23 |
|  | C14 | Dhe03 | 23, 237, 249 |
|  | C15 | Dje01 |  |
|  | C16 | Dje02 |  |
|  | C17 | Chcu01 |  |
|  | C18 | Chmd02 | 246 |
|  | C19 | Ddr01 |  |
|  | C20 | Dra01 |  |
|  | C21 | Dre01 | 152 |
|  | C22 | Dre02 |  |
|  | C23 | Ddr02 | 209 |
|  | C24 | Chla01, Chla02, Chla03 |  |
|  | C25 | Chzh01, Chzh02 | 60 |
|  | C26 | Chgu01, Dra02 |  |
|  | C27 | Ma9 | 314, 333 |
| **EASTERN** | E1 | UkraineA |  |
|  | E2 | RussiaB |  |
|  | E3 | SlovakiaA |  |
|  | E4 | SlovakiaB |  |
|  | E5 | PolandB, PolandC |  |
|  | E6 | Hungary, CZve04 | 23 |
|  | E7 | Finland |  |
|  | E8 | Avi01 | 102 |
|  | E9 | Avi02 | 118 |
| Lineages | Haplotypes | Samples | Amino acid positions |
| **EASTERN** | E10 | CZve01 |  |
|  | E11 | CZve02 | 60 |
|  | E12 | Dfu02 |  |
| **ITALIAN** | I1 | Italy |  |
|  | I2 | Chch03 | 209, 357 |
|  | I3 | Chch04 | 127, 209, 288, 357 |
|  | I4 | Chch01, Chch02 | 209 |
