# Supplementary material for: New Insight into the Colonization Processes of Common Voles: Inferences from Molecular and Fossil Evidence
Source: PLoS One. 2008 Oct 29;3(10):e3532. doi: 10.1371/journal.pone.0003532 (PMC2570793; doi:10.1371/journal.pone.0003532)
Supplement: Table S4 — Alternative topologies nonsignificantly different (5% confidence level) including the five lineages of Microtus arvalis. The topology in bold is the previously published topology [33], [34], while the topology in red corresponds to the present topology based on cytochrome b gene sequences (Figure 2). (0.09 MB DOC) [file pone.0003532.s004.doc]

**Table S4.** Alternative topologies nonsignificantly different (5% confidence level) including the five lineages of *Microtus arvalis*

The topology in bold is the previously published topology [33, 34], while the topology in red corresponds to the present topology based on cytochrome *b* gene sequences (Figure 2).

| Trees | -LnL^a^ | ∆ LnL^b^ | P > 0.05 |
| --- | --- | --- | --- |
| 1: ((((W,F),I),E),C); | 1668.72327 | best tree |  |
| 2: (((W,F),I),(C,E)); | 1668.72327 | 0.00000 | 1.000 |
| 3: ((((W,F),I),C),E); | 1668.72327 | 0.00000 | 0.999 |
| **4: (W,(F,((C,E),I)));** | **1668.72327** | **0.00000** | **0.993** |
| 5: (((W,F),(C,E)),I); | 1668.72327 | 0.00000 | 0.985 |
| 6: ((W,((C,E),I)),F); | 1668.72327 | 0.00000 | 0.978 |
| **7: ((W,F),((C,E),I));** | **1668.72327** | **0.00000** | **0.976** |
| 8: ((((W,F),C),E),I); | 1672.15977 | 3.43650 | 0.551 |
| 9: ((((W,F),C),I),E); | 1672.15977 | 3.43650 | 0.551 |
| 10: (((W,F),C),(E,I)); | 1672.15977 | 3.43650 | 0.551 |
| 11: (((W,F),(E,I)),C); | 1672.15977 | 3.43650 | 0.551 |
| 12: ((W,F),(C,(E,I))); | 1672.15977 | 3.43650 | 0.551 |
| 13: ((W,(C,(E,I))),F); | 1672.15977 | 3.43650 | 0.551 |
| 14: (W,(F,(C,(E,I)))); | 1672.15977 | 3.43650 | 0.551 |
| 15: (((W,F),(C,I)),E); | 1672.19469 | 3.47142 | 0.550 |
| 16: ((((W,F),E),C),I); | 1672.19469 | 3.47142 | 0.550 |
| 17: ((((W,F),E),I),C); | 1672.19469 | 3.47142 | 0.550 |
| 18: (((W,F),E),(C,I)); | 1672.19469 | 3.47142 | 0.550 |
| 19: ((W,F),((C,I),E)); | 1672.19469 | 3.47142 | 0.550 |
| 20: ((W,((C,I),E)),F); | 1672.19469 | 3.47142 | 0.550 |
| 21: (W,(F,((C,I),E))); | 1672.19469 | 3.47142 | 0.550 |
| 22: ((((W,I),F),C),E); | 1672.66816 | 3.94489 | 0.486 |
| 23: ((((W,I),F),E),C); | 1672.66816 | 3.94489 | 0.486 |
| 24: (((W,I),F),(C,E)); | 1672.66816 | 3.94489 | 0.486 |
| 25: (((W,I),(C,E)),F); | 1672.66816 | 3.94489 | 0.486 |
| 26: ((W,(F,(C,E))),I); | 1672.66816 | 3.94489 | 0.486 |
| 27: ((W,I),(F,(C,E))); | 1672.66816 | 3.94489 | 0.486 |
| 28: (W,((F,(C,E)),I)); | 1672.66816 | 3.94489 | 0.486 |
| 29: (((W,(F,I)),C),E); | 1673.05050 | 4.32723 | 0.460 |
| 30: (((W,(F,I)),E),C); | 1673.05050 | 4.32723 | 0.460 |
| 31: ((W,(F,I)),(C,E)); | 1673.05050 | 4.32723 | 0.460 |
| 32: (((W,(C,E)),F),I); | 1673.05050 | 4.32723 | 0.460 |
| 33: (((W,(C,E)),I),F); | 1673.05050 | 4.32723 | 0.460 |
| 34: ((W,(C,E)),(F,I)); | 1673.05050 | 4.32723 | 0.460 |
| 35: (W,((F,I),(C,E))); | 1673.05050 | 4.32723 | 0.460 |

^a^Log likelihood value for the best maximum-likelihood tree and the other evaluated topologies

^b^ Log-likelihood difference between the best ML tree and the evaluated topologies
