# Supplementary material for: New Insight into the Colonization Processes of Common Voles: Inferences from Molecular and Fossil Evidence
Source: PLoS One. 2008 Oct 29;3(10):e3532. doi: 10.1371/journal.pone.0003532 (PMC2570793; doi:10.1371/journal.pone.0003532)
Supplement: Table S5 — Primers used for PCR-amplification of the cytochrome b gene [28], [67] and the control region [66] (0.03 MB DOC) [file pone.0003532.s005.doc]

**Table S5.** Primers used for PCR-amplification of the cytochrome *b* gene [28, 67] and the control region [66]

| cytochrome *b* gene primers | |
| --- | --- |
| Foward primers |  |
| L7 | 5’–ACCAATGACATGAAAAATCATCGTT–3’ |
| L8 | 5’–CTGCCATGAGGACAAATATCATT–3’ |
| Reverse primers |  |
| H6 | 5’–TCTCCATTTCTGGTTTACAAGAC–3’ |
| H12 | 5’–GAAGAATCGTGTAAGGGTGGCTT–3’ |
| control region primers | |
| Forward primer |  |
| Pro+ | 5’-ACCATCAGCACCCAAAGCTG-3’ |
| Reverse primer |  |
| Mico4- | 5’-AGGTAAGAACCAGATGCCT-3’ |
