# Supplementary figures and images for: New Insight into the Colonization Processes of Common Voles: Inferences from Molecular and Fossil Evidence
Source: PLoS One. 2008 Oct 29;3(10):e3532. doi: 10.1371/journal.pone.0003532 (PMC2570793; doi:10.1371/journal.pone.0003532)

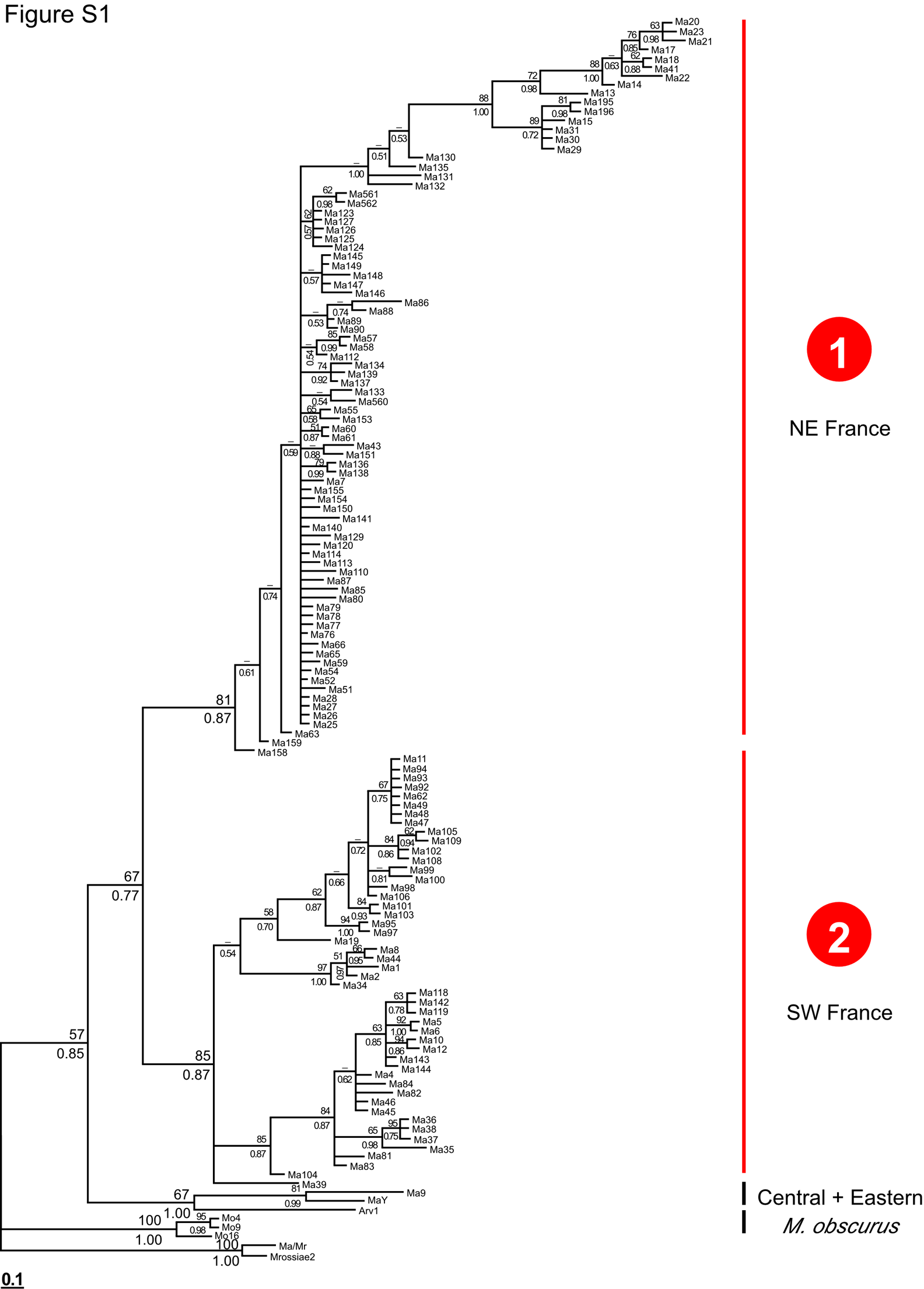

Supplement: Figure S1 — Bayesian tree reconstructed from control region sequences of Microtus arvalis. Individual labels are detailed in Table S1. The numbers at nodes refer to ML bootstrap percentages ≥50% (above branches) and BA posterior probabilities ≥0.50 (below branches). The five main evolutionary lineages as previously mentioned [34] are indicated on the right. (0.34 MB DOC) [file pone.0003532.s006.doc]
